# The Potential of TaqMan Array Cards for Detection of Multiple Biological Agents by Real-Time PCR

**DOI:** 10.1371/journal.pone.0035971

**Published:** 2012-04-23

**Authors:** Phillip A. Rachwal, Helen L. Rose, Victoria Cox, Roman A. Lukaszewski, Amber L. Murch, Simon A. Weller

**Affiliations:** Defence Science and Technology Laboratory, Porton Down, Salisbury, United Kingdom; National Taiwan University, Taiwan

## Abstract

The TaqMan Array Card architecture, normally used for gene expression studies, was evaluated for its potential to detect multiple bacterial agents by real-time PCR. Ten PCR assays targeting five biological agents (*Bacillus anthracis, Burkholderia mallei, Burkholderia pseudomallei, Francisella tularensis, and Yersinia pestis*) were incorporated onto Array Cards. A comparison of PCR performance of each PCR in Array Card and singleplex format was conducted using DNA extracted from pure bacterial cultures. When 100 fg of agent DNA was added to Array Card channels the following levels of agent detection (where at least one agent PCR replicate returned a positive result) were observed: *Y. pestis* 100%, *B. mallei & F. tularensis* 93%; *B. anthracis* 71%; *B. pseudomallei* 43%. For *B. mallei* & *pseudomallei* detection the BPM2 PCR, which detects both species, outperformed PCR assays specific to each organism indicating identification of the respective species would not be reproducible at the 100 fg level. Near 100% levels of detection were observed when 100 fg of DNA was added to each PCR in singleplex format with singleplex PCRs also returning sporadic positives at the 10 fg per PCR level. Before evaluating the use of Array Cards for the testing of environmental and clinical sample types, with potential levels of background DNA and PCR inhibitors, users would therefore have to accept a 10-fold reduction in sensitivity of PCR assays on the Array Card format, in order to benefit for the capacity to test multiple samples for multiple agents. A two PCR per agent strategy would allow the testing of 7 samples for the presence of 11 biological agents or 3 samples for 23 biological agents per card (with negative control channels).

## Introduction

The Polymerase Chain Reaction (PCR) is commonly used to detect pathogens from various sample types [1], [2]. A PCR assay asks a biased question, i.e. are you agent X? To act as a screening capability, where one sample is analysed by a panel of PCR assays, PCRs must either be multiplexed where multiple reactions are carried out in a single tube [3], [4], or an engineering solution such as microfluidics [5] or robotics [6] be found. The ability of conventional single-tube real-time PCR systems to act as a screening capability is limited by factors such as number of available reaction chambers, operative burden, and amount of DNA extract available to analyse.

Gene expression arrays, based on PCR, have been developed to analyse cDNA generated from an RNA template [7], [8]. This cDNA is generally added to the array at the nanogram (ng) scale and individual PCR assays report on the expression of individual genes. Direct, low-level, detection of a bacterial agent, with bacterial genomes known to have a weight at the single figure femtogram (fg) scale, would therefore require detection of genetic material at levels some six orders of magnitude lower than that required for the analysis of gene expression.

A recent study [9] reported the application of TaqMan Array Cards for the simultaneous detection of 21 respiratory agent pathogens. In this paper we report on the development and evaluation of the same array architecture for the detection of bacterial agents capable of causing acute disease. Ten PCRs were taken from existing research programmes and placed onto TaqMan Array cards. PCR performance on the array architecture and also in a standard singleplex PCR format was compared.

## Materials and Methods

### Bacterial DNA used in this study

DNA extracted and purified from *Bacillus anthracis* (Ames), *Francisella tularensis* (Schu4), and *Yersinia pestis* (CO92) was obtained from the Critical Reagents Program (CRP), MD, USA. *Burkholderia mallei* (NCTC 10230) DNA was obtained from the National Collection of Type Cultures (NCTC), Health Protection Agency, Porton Down, UK. *B. pseudomallei* (CLO2) DNA was obtained from Defence Science and Technology Organisation (DSTO), Melbourne, Australia. Due to the difficulties in accurately quantifying DNA at the femtogram scale DNA concentrations, as provided at source, were regarded as putative. All DNA extracts were sterility checked to enable work under Biological Safety Level 2 (BSL2) conditions to be carried out.

The definition of genome equivalent (GE), the weight of one bacterial genome in fg, was determined for each of the five agents by using the following values; Weight of a Dalton = 1.66×10^−24^ g; average molecular weight of a nucleotide base pair = 660 Da. The size, in base pairs, of each agent genome was then obtained from an online resource (http://cmr.jcvi.org/cgi-bin/CMR/shared/Genomes.cgi?crumbs=genomes) and the following calculation performed (where *n* = genome size in bp) to determine the weight of each genome:
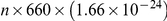



### Real-time PCR assays used in this study

Ten PCR assays used in this study are summarised in Table 1. The development and validation of the *F. tularensis* PCRs has been described previously [1]. The development and validation of the PCR assays for *B. anthracis*, *B. mallei*, *B. pseudomallei*, and *Y. pestis* will be described elsewhere. All PCRs have been shown to be specific for their target agents with the exception of the *Y. pestis* mgbA assay which has been shown to also detect *Y. pseudotuberculosis* (data not shown).

**Table 1:** Nucleotide sequences of PCR primers and probes used in this study.

Target	PCR	Sequences (5′-3′)	Array Card Probe	Singleplex Probe
*B. anthracis*	pXO1 MGB	F: CATTAAAGTTTTGGCCTGTATAGTCAA		
(pXO1)		R: GGATTTGCAGAAGGAATGGAAA		
		Pr: CTGCCACCCTTCG	FAM-MGB^a^	FAM-MGB
*B. anthracis*	pXO2 MGB	F: CGCTGGCGCTTCAATTCT		
(pXO2)		R: AGAGATGACAAAGCAAGGGATGA		
		Pr: CCTGCTTTCACTGCTT	FAM-MGB	FAM-MGB
*B. mallei* &	BPM2	F: CACCGGCAGTGATGAGCCAC		
*B. pseudo.*		R: ATGCTCCGGCCTGACAAACG		
(chr)		Pr: ACGCCCGTCGAAGCCCGAATC	FAM-TAM	FAM-BHQ1
*B. mallei*	BMI22	F: CAATGGCCCACAGGATCAG		
(chr)		R: GAACGACTATGAGGCCATTCAGT		
		Pr: CGTTGCGGCAGACTCGTGCAA	FAM-TAM	FAM-BHQ1
*B. pseudo.*	BPSCTN2	F: GTCAAGAATGCCGCGTACTTC		
(chr)		R: CGATCACCGCGCTCTATACC		
		Pr: CAGGATCACTGCCGCTCTCGT	FAM-TAM	FAM-BHQ1
*F. tularensis*	FTT0376	F: CCATATCACTGGCTTTGCTAGACTAGT		
(chr)		R: TGTTGGCAAAAGCTAAAGAGTCTAAA		
		Pr: AAATTATAAAACCAAACCCAGACCTTCAAACCACA	FAM-TAM	FAM-BHQ1
*F. tularensis*	FTT0523	F: ATGCCAAGTCTTTCACAACCAA		
(chr)		R: CTTTTGAATACGCACAGCTATATGG		
		Pr: AAAAAGCATCTTAATACCTCTACCACCACTAAAAATCCAA	FAM-TAM	FAM-BHQ1
*Y. pestis*	pPCP MGB	F: CGGCAATCGTTCCCTCAA		
(pPCP)		R: GGTCAGGAAAAAGACGGTGTGA		
		Pr: AACCATGACACGGTAGACT	FAM-MGB	FAM-MGB
*Y. pestis*	pMT MGB	F: GCGTCATCGGCAAAACCT		
(pMT)		R: AGGCGGAAAAGGCAAACAG		
		Pr: AGCACTTGCGCTAGC	FAM-MGB	FAM-MGB
*Y. pestis*	mpbA MGB	F: CGTCGCAGGGAGCAAAA		
(chr)		R: TGCATGCCGGATTAAGCTATG		
		Pr: CGACCCACTCGGTAGA	FAM-MGB	FAM-MGB

a Reporter/Quencher (5′-3′) dye combination of each probe used in this study.

### PCRs on TaqMan Array Cards

Each PCR assay (forward and reverse primers, probe) was placed within the TaqMan Array Card (Life Technologies Corporation, Foster City, CA, USA) architecture by the manufacturer. Cards comprised 8 channels of 24 individual reactions. Each PCR reaction comprised two replicates providing 48 individual (1 µL) reaction chambers per sample and 384 reactions per card. The 13 additional PCR reactions in each channel, plus the manufacturers card control PCR, were filled with assays required for an alternate study. Assays were deposited onto the cards by the manufacturer at the 9×10^−7^ mol/L primer (forward and reverse) concentrations with the probe at the 2×10^−7^ mol/L.

Cards were loaded by mixing 50 µL DNA extract with 50 µL TaqMan Universal Master Mix II (Life Technologies Corporation). This reaction mix was then pipetted into the inlet port of each channel. Cards were centrifuged (2×1 min, 1200 g), sealed and the inlet ports removed following the manufacturer's instructions. Cards were run on the ViiA™ 7 real-time PCR system (Life Technologies Corporation) using PCR cycling conditions comprising 30 s at 60°C, 10 min at 95°C followed by 40 two-step cycles of 15 s at 95°C and 1 min at 60°C.

### PCRs in singleplex format

PCR primers and probes were purchased from two suppliers (ATD Bio Ltd, Southampton, UK; Life Technologies Corporation). Real-time PCRs (25 µL volume) comprised 12.5 µL TaqMan Universal Master Mix II (Life Technologies Corporation), primers (9×10^−7^ mol/L), probe (2×10^−7^ mol/L) and 5 µL template. PCRs were run in optical 96-well PCR plates on the 7500 real-time PCR system (Life Technologies Corporation) using the same cycling conditions as used for the TaqMan array cards. The *Burkholderia* and *F. tularensis* PCR probes were labelled with the 3′ BHQ-1 quencher dye for the singleplex work, unlike the equivalent PCRs on the array cards which were labelled with the 3′ TAMRA quencher dye by the array card manufacturer. We have assumed that this minor change to the reporting chemistry did not affect PCR performance.

### Linearity of real-time PCRs on TaqMan Array Cards

To determine if PCRs on the array cards retained linearity across a range of target concentrations five cards were loaded with decreasing amounts of DNA from each of the five agents. Two replicates of putative amounts of 1 nanogram (ng), 100 picograms (pg), 10 pg, and 1 pg were loaded into the channels of one card (one card per agent).

### Limit of detection studies of real-time PCRs in both TaqMan Array Card and singleplex format

For each of the five biological agents one card was loaded with a putative amount of 500 fg DNA per channel and two cards were loaded with a putative amount of 100 fg DNA per channel. Seven channels per card were loaded with this amount giving 7 replicates for the 500 fg amount and 14 replicates for the 100 fg amount. The eighth channel on each card was loaded with a mixture of 50 µL sterile H_2_O and 50 µL TaqMan Universal Master Mix II and acted as a negative control.

Each of the 10 PCRs was also run in the singleplex format. Twenty-eight PCR replicates (equivalent to the number of replicates of each PCR challenged with the 100 fg DNA amount on the Array Cards) were challenged with 100 fg of target agent DNA per PCR, and a further 28 replicates challenged with 10 fg DNA per PCR. The same DNA stocks were used for both the Array Card and Singleplex PCR experiments.

## Results

### Linearity of real-time PCRs on TaqMan Array Cards

**Figure 1:**
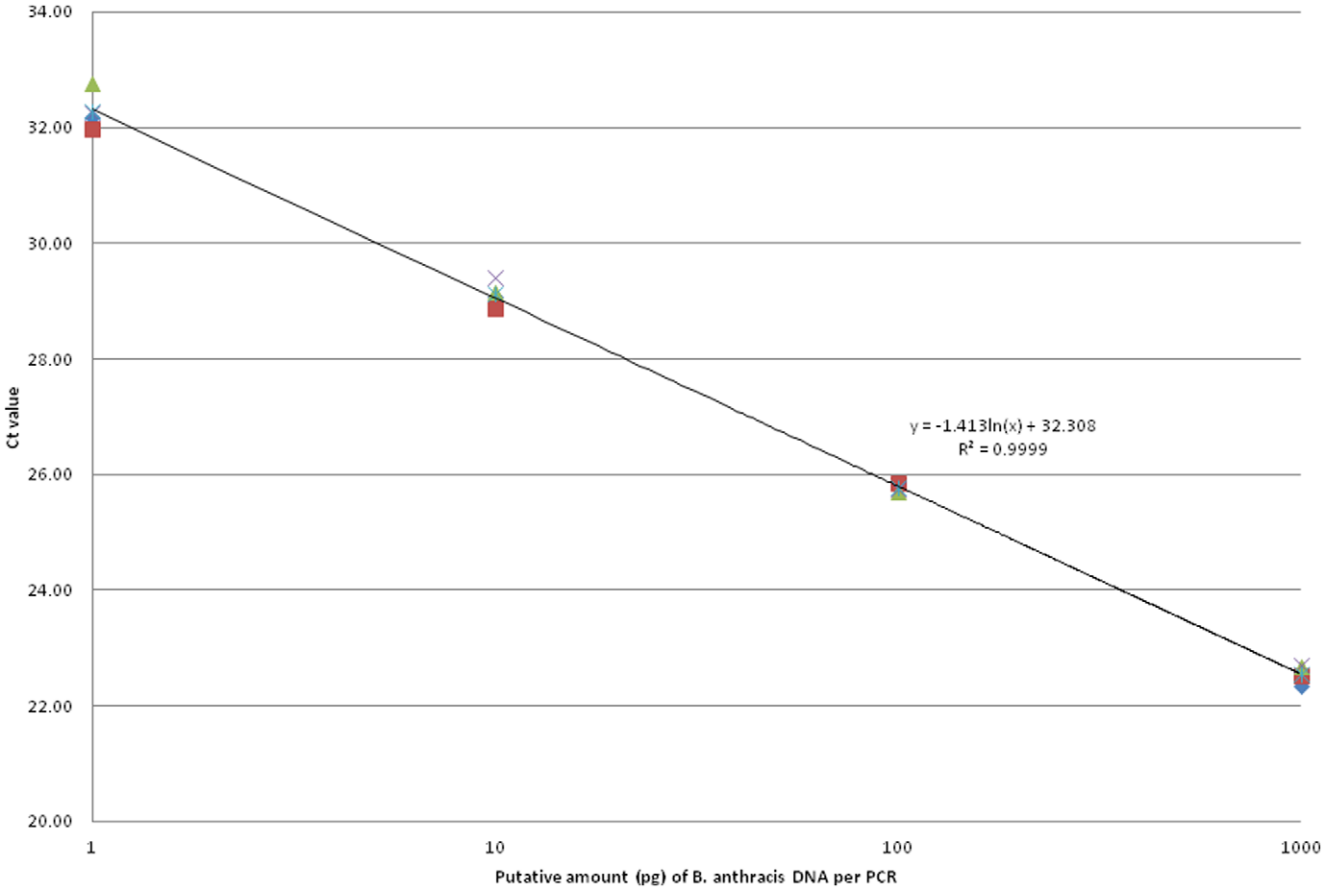
Quantification of B. anthracis (Ames) DNA by pXO2 MGB PCR replicates incorporated within the TaqMan Array Cards. Trendline and R2 value generated from mean CT values by Microsoft Excel.

All PCRs retained linearity across the range of 1 ng to 1 pg DNA per channel. R^2^ values for trendlines derived from mean C_t_ values were greater than 0.96 for all PCR reactions (data not shown). The standard curve for the performance of the pXO2 MGB assay on the Array Card is shown in Figure 1.

### Limit of Detection of real-time PCRs on the TaqMan Array Cards and in singleplex format

Results from the Array Card and singleplex PCR experiments are summarised in Table 2. PCRs in singleplex form were more sensitive than PCRs in the Array Card. The addition of 100 fg of DNA to all PCRs in singleplex format resulted in positive results at near 100% levels. Only at the 10 fg level did the singleplex PCRs result in less than 100% of all PCRs being positive. On Array Cards not all agent PCRs would respond when 500 fg of agent DNA was added to a channel. However, the presence of multiple replicates to agent PCRs increased the chances of agent detection where agent detection is defined as a least one agent PCR replicate in a channel returned a positive result. At this definition all agents were detected 100% of the time when 500 fg DNA was added to each channel (7 replicates). At the 100 fg level (14 replicates) *Y. pestis* was detected 100% of the time, *B. mallei* & *F. tularensis* 93% of the time, *B. anthracis* 71% of the time and *B. pseudomallei* 43% of the time. PCR results from an Array Card when putative amount of 100 fg of *Y. pestis* DNA was added to 7 channels are summarised in Table 3. A paired t-test for average detection rates between PCRs in Array Card and singleplex formats at 100 fg DNA per channel/reaction was conducted. The analysis (not shown) indicated a significant increase in singleplex PCR performance at the 95% confidence level for *B. anthracis*, *B. pseudomallei* and *Y. pestis* detection and at the 90% level for *B. mallei* and *F. tularensis* detection.

**Table 2:** Summary of PCR results from Array Card and Singleplex PCR experiments.

Target & Genome Weight^a^	PCR	Putative amount of DNA added to each Array Card channel or PCR tube					
		500 fg		100 fg		10 fg	
		+ve's^b^	Mean C_T_ c	+ve's	Mean C_T_	+ve's	Mean C_T_
	pXO1 MGB (Array Card replicates)	14/14	35.6 (0.6)	4/28	37.0 (3.3)	nt	nt
	pXO2 MGB (Array Card replicates)	12/14	33.8 (1.1)	9/28	33.7 (0.6)	nt	nt
*B. anthracis*	Overall *B. anthracis* detection rate by each Array Card channel^d^	**7/7**		**10/14**			
(Ames)	pXO1 MGB (Singleplex replicates)	nt	nt	27/28	35.9 (0.4)	20/28	38.4 (0.5)
5.72 fg	pXO1 MGB (Singleplex replicates)	nt	nt	28/28	34.6 (0.2)	15/28	36.0 (0.9)
	Overall *B. anthracis* detection rate by Singleplex PCR			**55/56**		**35/56**	
	BPM2 (Array Card replicates)	13/14	36.3 (1.6)	12/28	37.1 (1.0)	nt	nt
	BMI222 (Array Card replicates)	1/14	35.4	2/28	36.7 (0.1)	nt	nt
*B. mallei*	Overall *B. mallei* detection rate by each Array Card channel	**7/7**		**13/14**			
(NCTC 10230)	BPM2 (Singleplex Replicates)	nt	nt	28/28	32.7 (0.7)	23/28	36.9 (1.0)
6.39 fg	BMI222 (Singleplex Replicates)	nt	nt	25/28	37.1 (1.0)	4/28	37.8 (0.8)
	Overall *B. mallei* detection rate by Singleplex PCR			**53/56**		**27/56**	
	BPM2 (Array Card replicates)	8/14	37.9 (0.8)	7/28	37.7 (0.3)	nt	nt
	BPSCTN22 (Array Card replicates)	10/14	36.8 (1.2)	1/28	36.2	nt	nt
*B. pseudo-mallei*	Overall *B. pseudomallei* detection rate by Array Card channel	**7/7**		**6/14**			
(CLO2)	BPM2 (Singleplex replicates)	nt	nt	28/28	35.7 (0.4)	16/28	38.1 (0.6)
7.77 fg	BPSCTN2 (Singleplex replicates)	nt	nt	28/28	36.6 (0.2)	13/28	38.9 (0.5)
	Overall *B. pseudomallei* detection rate by Singleplex PCR			**56/56**		**29/56**	
	FTT0376 (Array Card replicates)	14/14	36.3 (1.0)	17/28	37.1 (0.7)	nt	nt
	FTT0523 (Array Card replicates)	13/14	37.5 (0.5)	9/28	38.4 (0.3)	nt	nt
*F. tularensis*	*Overall F. tularensis* detection rate by Array Card channel	**7/7**		**13/14**			
(Schu4)	FTT0376 (Singleplex replicates)	nt	nt	28/28	34.7 (0.4)	24/28	38.3 (0.5)
2.07 fg	FTT0523 (Singleplex replicates)	nt	nt	28/28	34.7 (0.1)	23/28	38.5 (0.6)
	Overall *F. tularensis* detection by Singleplex PCR			**56/56**		**51/56**	
	pPCP MGB (Array Card replicates)	12/14	33.4 (0.6)	10/28	32.7 (1.2)	nt	nt
	pMT MGB (Array Card replicates)	0/14	-	4/28	39.1 (0.7)	nt	nt
	mpbA MGB (Array Card replicates)	10/14	36.0 (0.3)	5/28	34.1 (0.5)	nt	nt
*Y. pestis*	Overall *Y. pestis* detection rate by Array Card channel	**7/7**		**14/14**			
(CO92)	pPCP MGB (Singleplex replicates)	nt	nt	28/28	34.6 (0.4)	15/28	37.1 (1.6)
5.28 fg	pMT MGB (Singleplex replicates)	nt	nt	28/28	37.0 (0.2)	3/28	39.2 (0.2)
	mpbA MGB (Singleplex replicates)	nt	nt	28/28	34.8 (0.4)	13/28	37.4 (0.3)
	Overall *Y. pestis* detection rate by Singleplex PCR			**84/84**		**31/84**	

a *B. mallei* genome calculation performed using ATCC 23344 genome size. *B. pseudomallei* calucalation using 1106B genome size. Other genome calculations performed using genome sizes of stated strains.

b No. of PCR positives from *n* replicates. nt - Not tested.

c C_T_ value: PCR cycle number at which fluorescence first detected. Mean of positives only. Variance of mean in paranthesis.

d Detection rate defined as at least one agent PCR positive replicate in the Array Card channel.

**Table 3:** Schematic representation of detection of 100 fg of *Y. pestis* DNA in seven Array Card channels across one Array Card.

PCR^a^	TaqMan Array Card and putative amount of DNA added per channel															
	Ch1-100 fg		Ch2-100 fg		Ch3-100 fg		Ch4-100 fg		Ch5-100 fg		Ch6-100 fg		Ch7-100 fg		Ch8-No DNA	
	Rep 1	Rep 2	Rep 1	Rep 2	Rep 1	Rep 2	Rep 1	Rep 2	Rep 1	Rep 2	Rep 1	Rep 2	Rep 1	Rep 2	Rep 1	Rep 2
PCR 1																
PCR 2																
**Ba pXO1 MGB**	#	#	#	#	#	#	#	#	#	#	#	#	#	#	#	#
PCR 4																
PCR 5																
PCR 6																
PCR 7																
PCR 8																
PCR 9																
**BMI22**	#	#	#	#	#	#	#	#	#	#	#	#	#	#	#	#
Card Control																
**BPSCTN2**	#	#	#	#	#	#	#	#	#	#	#	#	#	#	#	#
**BPM2**	#	#	#	#	#	#	#	#	#	#	#	#	#	#	#	#
PCR 13																
PCR 14																
**Ft FTT0376**	#	#	#	#	#	#	#	#	#	#	#	#	#	#	#	#
**Ft FTT0523**	#	#	#	#	#	#	#	#	#	#	#	#	#	#	#	#
PCR 17																
PCR 18																
PCR 19																
**pXO2 MGB**	#	#	#	#	#	#	#	#	#	#	#	#	#	#	#	#
**Yp pPCP MGB**	#	**33.28** b	#	**32.05**	**32.81**	#	**33.16**	**32.12**	#	**34.10**	#	**33.08**	#	**34.16**	#	#
**Yp pMT MGB**	#	#	#	#	**39.72**	**39.97**	#	#	#	#	#	#	#	#	#	#
**Yp mpbA MGB**	#	#	#	#	#	**34.83**	#	#	#	#	#	#	#	#	#	#

a Numbered PCR represents assays evaluated in an alternate study. Named PCRs this study.

b C_T_ value: PCR cycle number at which fluorescence first detected. # = negative result.

## Discussion

The architecture of the Array Cards indicated factors which may affect sensitivity. Firstly with the total volume of the sample being 100 µL (50 µL DNA extract and 50 µL PCR mastermix) and the channel comprising 48×1 µL reaction chambers not all the DNA in the sample would be analysed by a PCR assay. Secondly, a given PCR chamber will not necessarily contain a PCR assay targeting the agent DNA that has been added to the channel.

In addition at low concentrations target DNA sequences are known to be stochastically distributed [10] with the number of targets present in a given aliquot either varying or not even being present. One hundred fg of *B. anthracis* Ames DNA equates to approx. 17 Genome Equivalents (GEs). An equal distribution of *B. anthracis* DNA would therefore result in 0.35 GEs being deposited in each of the 48 reaction chambers in the channel - below the reproducible limit of detection of any PCR [11]. However, a stochastic distribution would mean that higher amounts of DNA would be placed in some chambers than others suggesting that the more assays (and therefore chambers) to one agent in a channel the more likely it would be that an agent be detected.

The results of our study confirmed our initial assumptions. We defined agent detection as at least one of the PCR replicates in a channel returning a positive result. At putative amounts of DNA from 500 fg per channel not all PCRs to the agent in question would return positive results – suggesting the stochastic distribution of DNA at low levels was observed by Array Card PCR assays from this level. For three of the five agents tested (*B. anthracis*, *F. tularensis*, and *Y. pestis*) overall detection rates were high at the 100 fg level (>70%) from the 14 replicates tested and 100% at the 500 fg level (7 replicates). When the same PCR assays were evaluated in singleplex format each PCR exhibited near 100% detection rates when 100 fg of DNA was added to each (25 µL) reaction. Only at the 10 fg was the stochastic distribution of DNA in the extracts observed in the singleplex format – in terms of a negative to positive ratio from 28 replicates. Therefore, and as also observed previously [9], PCRs in the singleplex format are generally a log more sensitive than the same assays in the Array Card format. Users would have to tolerate this reduction in sensitivity for the advantage of being able to screen for multiple agents when using PCR assay in the Array Card format.

The results of the *Burkholderia* PCR assays demand closer inspection. The BPM2 assay is a PCR designed and validated to be able to detect both *B. mallei* and *B. pseudomallei*, with the BMI222 and BPSCTN2 PCRs designed and validated for the specific detection of each respective agent. Using the above definition of detection at the 100 fg level *B. mallei* was detected in 13/14 replicates and *B. pseudomallei* was detected in 6/14 replicates. However, in both these cases the generic BPM2 assay performed more effectively than the specific assays. The BMI222 assay only produced a positive result in two of the 13 *B. mallei* positive replicates and the BPSCTN2 assay produced a positive result in one of the 6 *B. pseudomallei* positive replicates. These results suggest that a reproducible identification specific to *B. mallei* or *pseudomallei* would not be achievable at the 100 fg level with these assay combinations. The higher genome weight of each organism (when compared to the other three agents) may partially account for these results in terms of decreased PCR target copy number in 100 fg of DNA, (which may also account for the low detection rate of *B. pseudomallei* at the 100 fg level). At the 500 fg level, where approximately 65 GEs (500÷7.7) were added to each channel, the *B. pseudomallei* BPSCTN2 assay returned at least one positive PCR replicate from each of the 7 DNA extracts tested. In comparison, the BMI222 PCR only returned one PCR positive from the 7 extracts tested at the 500 fg level, where 500 fg contained approximately 78 GEs (500÷6.39). The results from the singleplex PCR comparison do suggest that BMI222 assay has a reduced sensitivity (25/28 PCR positives at 100 fg and 4/28 PCR positives at 10 fg) when compared with the majority of the other PCR assays. In practical terms these results could encourage the retention of the BPSCTN2 assay in a deployed card format but also indicate the need to replace the BMI222 assay.

We cannot account for the poor performance of the BMI222 assay on the Array Cards. PCRs were deposited into each reaction chamber with primer concentrations of 9×10^−7^ mol/L (each) by the manufacturer. As a singleplex PCR the BMI222 assay has had its primer concentrations optimised at different concentrations (3×10^−7^ mol/L F-primer; 9×10^−7^ mol/L R-primer)]. If it had been possible to obtain cards with optimised PCR primer concentrations this would potentially have increased BMI222 PCR performance (as well as overall Array Card PCR performance). The authors of the previous study [9] were able to obtain cards with PCR primers that varied in concentration and thus were apparently optimised.

The choice of appropriate PCR assay (and gene targets) for optimal Array Card performance was also illustrated by the results produced by the *Y. pestis* pMT PCR which did not perform well on the array cards (e.g. no positives from seven 500 fg DNA extract replicates). This is possibly due to a low copy number of the target pMT plasmid within the *Y. pestis* genome [12]. The diagnostic utility of the pMT plasmid has been questioned in other studies [13] though a virulent strain (Pestoidies F) of *Y. pestis* without the high copy number pPCP plasmid is also known to exist [14] so care must be taken before discarding an assay that may help cover all pathogenic strains. However, targets of known high copy number or which are present on elements such as plasmids which increase in copy number during infection [15] should be favoured on the Array Card format.

In this study we did not use all available PCR reaction chambers. Therefore the cards could be used to test for more than 5 agents. The previous study [9] reports that one channel could support the detection of 21 different respiratory pathogen strains. From the results of our study we would consider that at least 2 appropriate PCR assays per agent (giving 4 replicates PCR chambers) are required for robust detection. This potentially increases confidence in identification (two separate genomic targets per agent) and maximises the chance of detection (increased possibility of agent DNA being deposited into a reaction chamber with an agent PCR assay).

Our approach to the number of PCR assays to agents would allow for the detection of 11 different agents in one channel if 2 control PCR assays, e.g. for DNA extraction [16], were also included. Alternatively a further set of assays could be incorporated into an adjacent channel allowing detection of 23 different agents (if 100 µL of DNA extract was available to test). In this scenario three samples could be analysed, with the seventh and eighth channels on the cards acting as a negative control. We would also consider further testing of a sample (by singleplex PCR or another technology) to confirm a positive result. In this instance the cards overcome the inherent bias of PCR by allowing as many agents to be tested for as possible, whilst also allowing further, confirmatory, targets to be tested to achieve a high as confidence result as possible. However, all these considerations, and selection of appropriate controls, can only be the consideration of each user.

A multiplexed PCR for the detection of *B.* anthracis, *F. tularensis* and *Y. pestis* has recently been described [3] with limits of detection lower than the detection limits for Array Card PCRs to same agents reported in our study. However, it is unlikely that a multiplexed real-time PCR assay could support the detection of 23 agents (with 2 real-time PCR assays per agent) in such a format due to factors such as individual reaction efficiencies and the availability of the required number of different fluorescent reporter dyes. Therefore the microfluidic architecture of the Array Cards, with individual reactions spatially separated, allows for an increased number of agents to be tested for. The spatial separation also allows assays to be easily changed without the need for extensive re-optimisation and validation of a highly parallel multiplex assay. This could aid the rapid deployment of PCR assays to novel or emerging pathogens.

The small size of the reaction chambers on the cards (1 µL) underscores the importance of delivering as much target DNA to each chamber as possible. DNA extraction methods from larger sample volumes (mL rather than µL) than generally accounted for in current DNA extraction methods have been described [17]. This could deliver a more concentrated DNA extract to the Array Card channels. Additionally in our study samples added to each channel constituted 50 µL of DNA extract and 50 µL of 2× PCR mastermix. This ensures that 0.5 µL of extract was delivered to each PCR chamber. In the previous study [9] the volume of DNA extract added to each sample mix was 20 µL, ensuring that 0.2 µL of extract was delivered to each chamber. The use of a 4× PCR mastermix would allow 75 µL of extract to be added to each channel, ensuring 0.75 µL of extract was delivered to each chamber. These alterations to method could potentially increase the sensitivity and reproducibility of assays in the Array Card format.

Despite the inherent, architectural, factors that induce a 10-fold loss in sensitivity of Array Card PCR assays, the previous study [9] achieved an 89% detection rate when using Array Card PCRs, compared to the same PCRs in singleplex format, when testing 292 clinical samples for respiratory agents. This study therefore indicates that, with an appropriate DNA extraction method, the gap in sensitivities between Array Card and singleplex PCRs can be bridged. Any DNA extraction method selected should account for factors such as increased levels of background DNA and residual PCR inhibitors affecting the performance of the Array Card PCRs. These factors may be exacerbated by the small reaction volume of each PCR chamber (1 µL) on the Array Cards, when compared to a singleplex PCR volume of 25 µL. Future work will determine the performance of our Array Card PCR system on samples derived from murine infection models in an attempt to answer this question.

The customised array card architecture was evaluated under BSL2 conditions. DNA from Biological Safety Level 3 organisms was analysed. The DNA extracts tested were sterility checked (plating out 10% of DNA extract onto microbiological media and confirming the absence of bacterial growth), enabling use of the DNA at BSL2 conditions. This process took a period of days. For routine detection of BSL3 organisms this process may not allow identification by the time required for the result to be actionable (e.g. administration of medical countermeasures). The size of the Array Card Viia7 reader (Dimensions 53.3×63.5×64.5 cm) would also preclude it being placed in all but the largest Microbiological Safety Cabinets (MSC), notwithstanding the additional requirement for a centrifuge. Some DNA extraction methods [2] heat samples (e.g. 95°C; 15 mins) in the presence of chemical lysis buffers containing ingredients (guanidine salts) with known bactericidal efficacy [18]. The crude DNA extract is then purified with ethanol based wash buffers. Depending on the nature and context of the sample it may be that such methods could be verified to ensure that DNA extracts coming directly out of a BSL3 cabinet do not contain viable organisms and could be tested by the Array Card format without the need to conduct a sterility check by microbiological plating. These practical considerations must be taken into account if the Array Cards were to be used for routine pathogen screening.
